# Prevalence study of *Echinococcus granulosus* in guanaco (*Lama guanicoe*) from the Chilean Patagonia unexpectedly reveals the southernmost limit of *Taenia omissa*

**DOI:** 10.1017/S0031182024001501

**Published:** 2024-12

**Authors:** Cristian A. Alvarez Rojas, Juan Francisco Alvarez, Juliana Iglesias, Anson V. Koehler, Cristian Bonacic

**Affiliations:** 1 Escuela de Medicina Veterinaria, Facultad de Agronomía y Sistemas Naturales, Facultad de Ciencias Biológicas y Facultad de Medicina, Pontificia Universidad Católica de Chile, Santiago, Chile; 2 Servicio Agrícola y Ganadero, Región de Magallanes, Chile; 3 Faculty of Science, The University of Melbourne, Parkville, VIC 3010, Australia; 4 Departamento de Ecosistemas y Medio Ambiente, Facultad de Agronomía y Sistemas Naturales, Pontificia Universidad Católica de Chile, Santiago, Chile

**Keywords:** Chile, co-infection, *Echinococcus granulosus*, guanaco, Patagonia, *Taenia omissa*

## Abstract

This study presents the first detection of *Taenia omissa* metacestodes in guanaco (*Lama guanicoe*) within the Chilean Patagonia, marking the southernmost record of natural infection in an intermediate host on the continent. *Taenia omissa* was found in the continental part of the Magallanes region where the top predators are pumas (*Puma concolor*). Conversely, all metacestodes found in guanacos collected from Tierra del Fuego Island, where no pumas exist, were identified solely as *Echinococcus granulosus sensu stricto*. Additionally, this research highlights a tissue preference of *T. omissa* for liver, contrasting with *E. granulosus*, which predominantly affects the lungs in guanacos. We also report the infection of *T*. *pisiformis* in 1 guanaco. Our findings emphasize the need for accurate identification of metacestodes during meat inspection in an area where *T. omissa* and *E. granulosus* overlap. This research also contributes to increase the knowledge of parasite–host dynamics in wildlife and underscores the importance of considering broader spectrum intermediate hosts in the epidemiology of parasitic infections.

## Graphical abstract

**Figure:**
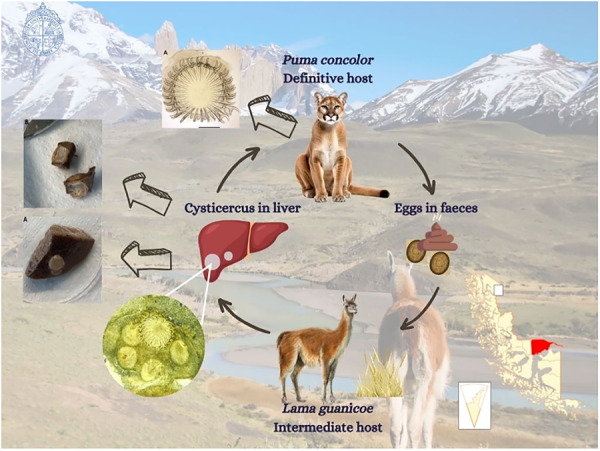

## Introduction

The tapeworm *Echinococcus granulosus*, responsible for cystic echinococcosis, is highly endemic in the Magallanes region in southern Chile (Alvarez Rojas *et al*., 2016; Deplazes *et al*., 2017). This area, characterized by its archipelago extending to the west and south, along with its mountainous terrain and elements of the Patagonian Ice Sheet, hosts the largest sheep population at national level, of approximately 2 million. Such a demographic provides ideal conditions for the transmission of *E*. *granulosus*, with canids serving as the definitive hosts. Recent data indicate a prevalence of 2.9% in sheep (Alvarez Rojas *et al*., 2016) and between 1.8 and 18% in dogs (Alvarez *et al*., 2021; Eisenman *et al*., 2023) across various areas of Magallanes. In this landscape, guanacos (*Lama guanicoe*), co-inhabiting with sheep on expansive private grasslands known as ‘Estancias’, are also susceptible to *E*. *granulosus* infection. Reports from the 1980s and 1990s showed prevalence rates between 3.1 and 12% in guanacos (Valdebenito Díaz, 2008; Carmanchahi and Lichtenstein, 2022).

Following a decline in the 1970s and 1980s, the guanaco population has rebounded to over 150 000 animals in Magallanes (WCS, 2022). The legalization of seasonal guanaco hunting since 2003 has promoted sustainable resource utilization, contributing to a growing trade in guanaco meat, leather and fibre. During meat inspection of hunted guanaco, the parasitic lesions in liver and lungs have been attributed to *E*. *granulosus* at meat inspection (SAG, 2022). However, some metacestodes, initially presumed to be *E*. *granulosus*, were morphologically identified as cysticercus of *Taenia* spp. This study aims to clarify the identity of these metacestodes, identify the *Taenia* species infecting guanacos and estimate the prevalence of both *Taenia* spp. and *E. granulosus* in guanacos from Chilean Patagonia.

## Materials and methods

### Sample collection

**Figure 1.**
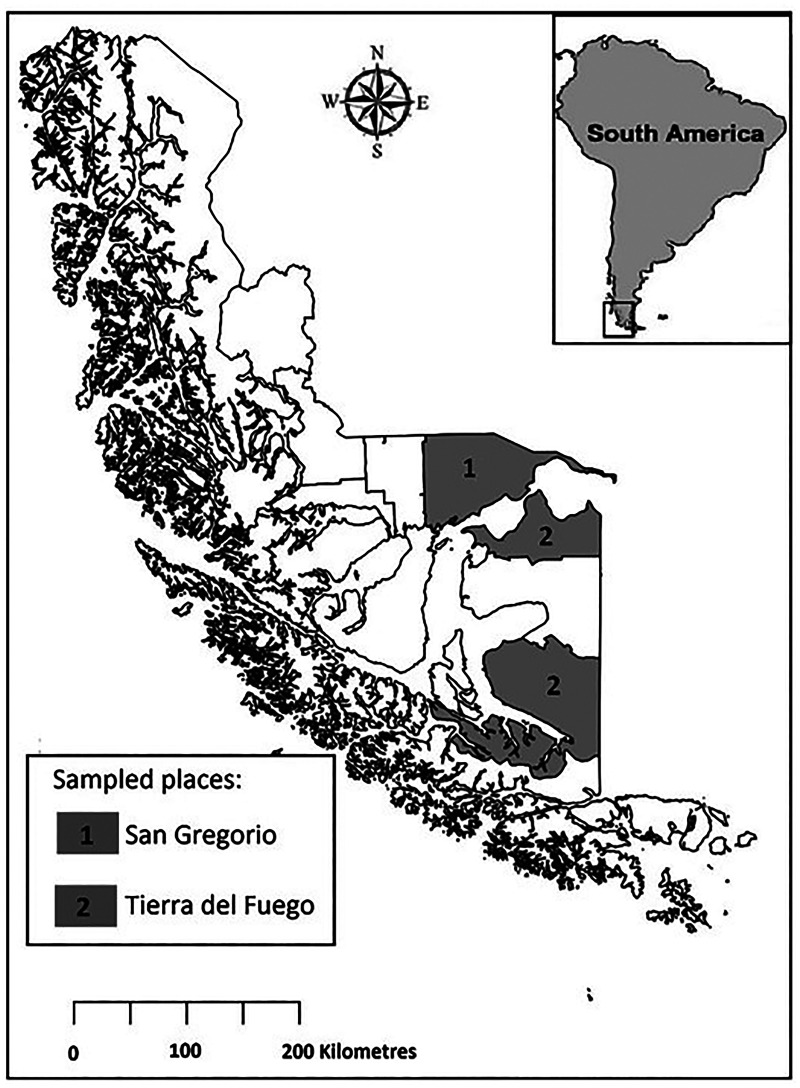
Map of the Magallanes region showing the locations where guanacos were captured, 1: San Gregorio in the continental part of the region and 2 in Tierra del Fuego Island divided in the north of the island Primavera and in the south of the island Timaukel.

During the year 2022, a total of 2704 animals were legally hunted in the Magallanes region: 36.8% (996 animals) from San Gregorio in the continental part of the region and 63.2% (1708 animals) in Tierra del Fuego Island (Fig. 1). Sex was recorded and the age of guanacos was estimated following dental examination (Kaufmann *et al*., 2017). Out of 2704 inspected carcasses, 73 animals (2.7%) showed parasitic lesions in liver and lungs initially attributed to *E*. *granulosus*. Out of 73 guanacos, 37 animals (50.6%) were hunted in San Gregorio and 36 animals (49.3%) in Tierra del Fuego. For molecular analysis we collected metacestodes from 56 guanacos: 27 from San Gregorio and 29 from Tierra del Fuego. Larval stages of cestodes were externally measured and the presence or absence of protoscolices of *E*. *granulosus* or a single scolex in the case of *Taenia* spp. was verified through microscopic examination of the metacestode content.

### DNA extraction and *cox1* PCR

DNA was extracted from each metacestode with the EZNA^®^ Tissue DNA Kit (Omega Bio-Tek) following manufacturer instructions. An initial PCR was performed to differentiate between *Taenia* spp. (12S rDNA, 267 bp) and *E*. *granulosus* (12S rDNA, 117 bp) using the primers Cest3, Cest4 and Cest5 designed by Trachsel *et al*. (2007). DNA tested positive for *Taenia* spp. underwent a second PCR for the amplification and sequencing of a section of the *cox1* gene (366 bp) (Bowles *et al*., 1992) to identify the species through comparison with GenBank entries. Both PCR were performed using Green GoTaq Master mix (Promega). The thermocycler conditions were 95°C for 2 min, and 35 cycles at 95°C for 30 s, 55°C for the initial PCR (Trachsel *et al*., 2007) and 50°C for *cox1* (Bowles *et al*., 1992) for 30 s and 72°C for 30 s. The PCR products were visualized through electrophoresis in 1.5% agarose and sequenced bidirectionally using the same primers (Macrogen, Chile).

### Sequence alignments, phylogenetic and haplotype analyses

Sequences of the *cox1* gene region were trimmed (320 bp) and aligned with those of representative *T. omissa* along with *T. hydatigena* as the outgroup using Muscle (Edgar, 2004). Phylogenetic analysis of the alignment was conducted using the Maximum Likelihood method in the program MEGA11 (Tamura *et al*., 2021) with initial model selection performed in IQ-TREE (Kalyaanamoorthy *et al*., 2017). Evolutionary distances were computed using the Hasegawa–Kishino–Yano (HKY) method with invariant sites. Each site, including gaps, were considered with a total of 2000 bootstrap replicates. The identification of haplotypes and the network analyses were computed with PopArt (Leigh and Bryant, 2015).

### Statistical analysis

Statistical analyses were performed to assess differences between parasite species and infection rates. An independent sample *t*-test was used to compare the sizes of *E. granulosus* and *T. omissa* metacestodes. Fisher's exact test was applied to evaluate the distribution of parasites across different geographic regions. Additionally, *χ*^2^ tests were conducted to examine the relationship between sex and infection rates.

## Results

### Genetic characterization of metacestodes

The first PCR results, performed on DNA isolated from 27 metacestodes collected in San Gregorio, identified *Taenia* spp. (267 bp) in 22 samples whereas 4 were identified as *E*. *granulosus* (117 bp); no amplification was achieved in 1 sample. Of the 22 isolates identified as *Taenia* spp., PCR amplification of the *cox1* gene was successful in all samples. However, only 20 samples produced a strong band enabling high-quality sequencing. Among these, 19 were identified as *T*. *omissa* and *T*. *pisiformis* (Table 1). Unique individual sequences of the *cox*1 gene from *T*. *omissa* acquired in this study were deposited in GenBank under accession numbers PP326076–PP326081. For Tierra del Fuego, PCR analysis, carried out on 29 metacestodes showed a band of 117 bp corresponding to *E*. *granulosus* in all samples. Sequencing of these PCR products showed 100% homology with *E*. *granulosus sensu stricto* (Table 1). No amplification was observed with primers targeting *Taenia* spp. DNA in samples from these areas.

**Table 1.** Distribution of *T. omissa* and *E. granulosus* in animals from San Gregorio in the continent and Tierra del Fuego Island from the Magallanes region in Chile

		Trachsel PCR			Sequencing *Taenia* spp. samples		
Locality	Number of animals	*E. granulosus*	*Taenia* spp.	Negative	*T. omissa*	*T. pisiformis*	No sequence
San Gregorio	27	4	22	1	19	1	2
Tierra del Fuego	29	25	0	4			

Numbers show the animals infected. Statistical analysis *via* Fisher's exact test confirmed significant differences in the distribution of both parasites between the 2 provinces.

### Prevalence of *T. omissa* and *E. granulosus*

We identified notable differences in the prevalence of *T. omissa* and *E. granulosus* infections between San Gregorio and Tierra del Fuego (Table 1). For San Gregorio, *T*. *omissa* was found in 73.1% of animals with metacestode lesions for which molecular identification was possible (19/26animals), whereas *E. granulosus* was identified in 15.4% (4/26) and *T*. *pisiformis* in 3.8% (1/26). Conversely, Tierra del Fuego showed a remarkable pattern, where all examined animals (25/25) were infected with *E. granulosus*.

### Biological features of metacestodes from guanaco

**Figure 2.**
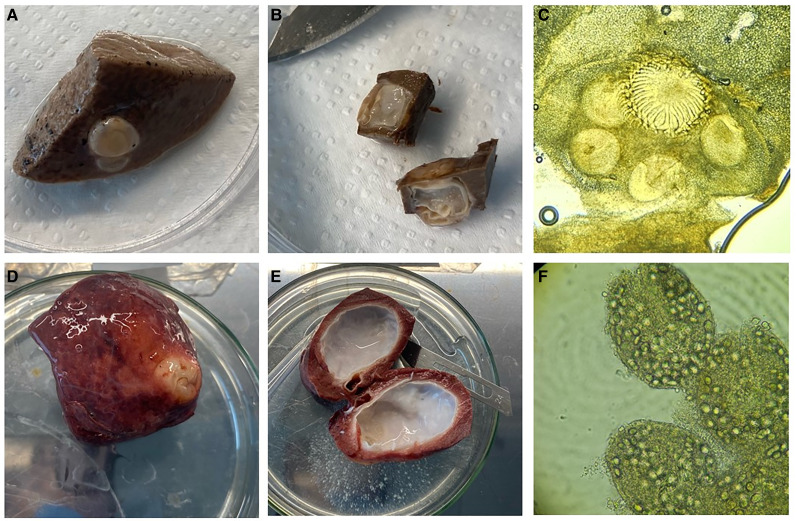
(A) *Taenia omissa* sample collected from liver tissue measuring 6 mm. (B) *Taenia omissa* cysticercus open showing internal structure. (C) Microscopic observation (40×) of the scolex of a cysticercus of *T. omissa* collected in this study. (D) *Echinococcus granulosus* metacestode collected from the lung of a guanaco measuring 38 mm. (E) *Echinococcus granulosus* cyst open showing internal structure and ‘hydatid sand’ on the Petri dish. (F) Protoescolices of *E. granulosus* observed microscopically (40×) in fertile cysts collected in this study.

The external aspect of a *T*. *omissa* cysticercus in liver tissue of a guanaco and a hydatid cyst of *E. granulosus* in the lung are shown in Fig. 2A and D respectively. Figure 2B and C shows the internal aspect of *T*. *omissa* cysticercus and a scolex of *T*. *omissa* from the same cyst, respectively. Figure 2E and F shows the internal aspect of a hydatid cyst and protoscolices from the same cyst, respectively. The average size of metacestodes was 8.72 mm (s.d. = 2.80 mm, range = 4.0–13.0 mm) for *T. omissa* and 21.88 mm (s.d. = 13.72 mm, range = 5.0–54.0 mm) for *E. granulosus*. *Echinococcus granulosus* parasites are significantly larger than those of *T. omissa* (*t*-statistic of −3.02 and a *P* value of 0.0049). While there is no significant difference in the age of guanacos infected with these 2 parasites (*P* value of 0.1305). Among the 29 cysts identified as caused by *E. granulosus* (4 from San Gregorio and 25 from Tierra del Fuego) protoscolices were observed in 10 samples, all of which were located in the lungs. None of the 12 cysts of *E. granulosus* found in the liver of a guanaco were fertile. Eight of the fertile cysts were found in male and only 2 in female guanacos.

No significant difference in *T*. *omissa* infection rates between females and males was found (*χ*^2^ statistic = 0.0, *P* value = 1.0) while a significant difference was observed in *E*. *granulosus* infection rates between sexes (*χ*^2^ statistic = 7.5625, *P* value = 0.006), suggesting that male guanacos are significantly more likely to be infected with *E*. *granulosus* than females (Table 2).

**Table 2.** Number of guanacos divided by sex infected with *T. omissa* and *E. granulosus* in places where samples were collected

	Number of animals infected with *T*. *omissa*			Number of animals infected with *E*. *granulosus*		
Province	Number of animals	Number of female	Number of male	Number of animals	Number of female	Number of male
San Gregorio	19	9	10	4	1	3
Tierra del Fuego	0	0	0	25	10	15
Total	19	9	10	29	11	18

In assessing the distribution of *E. granulosus* and *T. omissa* across liver and lung organs (Table 3) a *χ*^2^ test of independence indicated a statistically significant difference in the prevalence of these species between the 2 organs [*χ*^2^ (1) = 11.96, *P* < 0.001]. This suggests that the distribution of *E. granulosus* and *T. omissa* is not random across the liver and lungs, highlighting potential biological or ecological preferences in their organ colonization with *T*. *omissa* found more commonly in liver and *E*. *granulosus* in lungs of infected guanacos.

**Table 3.** Distribution of *E. granulosus* and *T. omissa* across metacestodes found in liver and lungs in guanacos

Organ	*E. granulosus*	*T. omissa*	*T. pisiformis*	*Taenia* spp.	Unidentified	Total
Liver	8	15	0	3	4	30
Lungs	21	4	1	–	–	26
Total	29	19	1	5	1	56

Cysts identified as *Taenia* spp and a single metacestode that could not be identified are also included.

### Phylogenetic analysis of *T. omissa*

**Figure 3.**
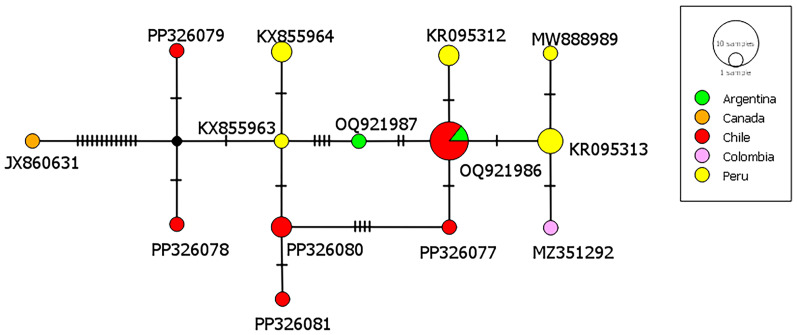
Haplotype network with the cox1 sequences from *Taenia omissa* in the present study and all similar sequences deposited in GenBank. The nucleotide diversity: *π* = 0.015875, Tajima's *D* statistic: *D* = −0.891768, *P* (*D* ≥ −0.891768) = 0.402249.

**Figure 4.**
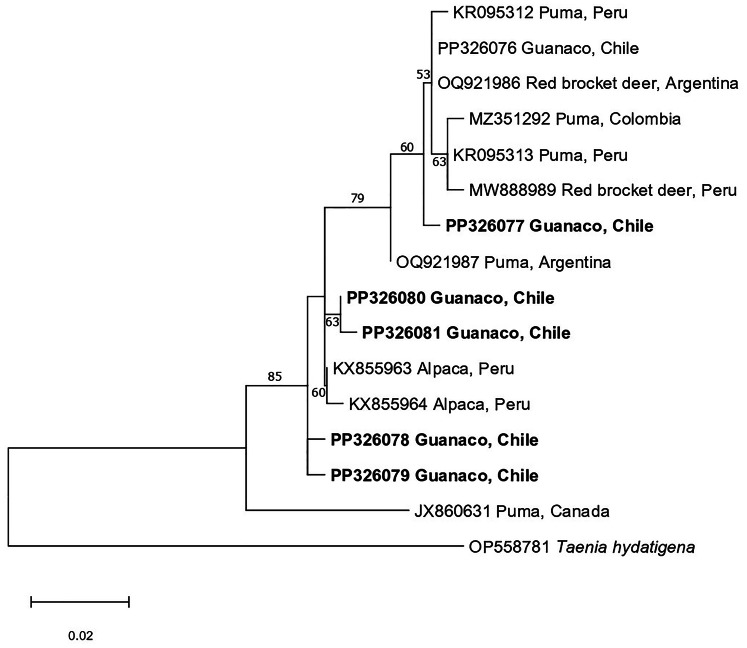
Relationship of the novel *Taenia omissa* taxa (in bold) from metacestode tissue found in guanacos with representative *T. omissa* sequences from GenBank, based on a phylogenetic analysis of sequence data from a portion of the mitochondrial cytochrome c oxidase 1 gene (cox1) employing the Maximum Likelihood method. Branch supports are represented by Maximum Likelihood bootstrap percentages. *Taenia hydatigena* was used as an outgroup.

For comparative purposes, all *cox*1 sequences acquired in this study and similar *T*. *omissa* sequences in GenBank were trimmed to 320 bp. From the 19 sequences identified as *T*. *omissa* we found 6 different haplotypes. One of these haplotypes was previously described in Argentina (OQ921986) while the other 5 variants had not been identified before. Further analysis revealed 25 segregating sites and 9 parsimony-informative sites when these sequences were compared with 11 sequences from GenBank, representing the same gene section of *T*. *omissa* from Argentina, Canada, Colombia and Peru. The haplotype network (Fig. 3) has a disperse distribution with none of the variants being the centre indicating a considerable genetic diversity among the *T*. *omissa* haplotypes. The phylogenetic tree (Fig. 4) illustrates that the *T. omissa* sequences extracted from Chilean guanacos cluster together with sequences from other parts of South America. Notably, these sequences form a distinct group separate from the sequence derived from a Canadian puma (JX860631).

## Discussion

This study provides the first evidence of *T. omissa* metacestodes in guanacos from the Chilean Patagonia, establishing a new southern limit for the parasite's distribution. *Taenia omissa* is known circulating between pumas and wild deer in North America ranging from white-tailed deer in Texas (Stubblefield *et al*., 1987) and Florida (Forrester and Rausch, 1990) to red brocket deer (*Mazama americana*) (Gomez-Puerta and Mayor, 2021; Arrabal *et al*., 2023) and alpacas (Gomez-Puerta *et al*., 2017) in South America. The present study shows a natural infection of guanacos as intermediate hosts, extending the known range of *T*. *omissa* over 4000 km south to Patagonia. This is a significant leap from its previous southern limit in Argentina's Iguazu National Park (25.7 SL) to Torres del Paine in Patagonia (50.9 SL) (Arrabal *et al*., 2023). The distribution of puma ranges from northern British Columbia to southern Magellan strait in Patagonia. It is not present in Tierra del Fuego Island. Puma is the single known definitive host of *T*. *omissa* (Rausch *et al*., 1983; Forrester and Rausch, 1990). Adult specimens of *T*. *omissa* have been reported from the intestine of pumas from Oregon (Rausch *et al*., 1983), southern Florida (Forrester *et al*., 1985), Texas (Waid and Pence, 1988) and Washington (Rickard and Foreyt, 1992). In the southern hemisphere, adults of *T*. *omissa* have been found in pumas from Paraguay (Schmidt and Martin, 1978), Brazil (Benatti *et al*., 2021), the Peruvian Highlands in the Cuzco region (Gomez-Puerta *et al*., 2016) and the Atlantic Forest in Argentina (Arrabal *et al*., 2023).

The detection of *T*. *omissa* in Chilean Patagonia contrasted with its absence in Tierra del Fuego, where pumas are absent providing insight into the complex transmission dynamics of this cestode, offering an ecological perspective on the distribution of its definitive host in low-density or cryptic habitats. Currently, no pumas have been observed on Tierra del Fuego Island; only feral domestic cats have been reported (Schuttler *et al*., 2018) This aligns with the results of the present study, underscoring the role of *T*. *omissa* as a potential bioindicator for puma presence. This insight is particularly valuable in understanding puma ecology, its interactions with prey like the guanaco (Fernández and Baldi, 2014) and informing conservation strategies in these regions.

The distinct organ tropism exhibited by *T*. *omissa*, favouring the liver, in contrast to *E*. *granulosus*, which predominantly infects the lungs, introduces an additional layer of intricacy to the differential diagnosis of cestode infections in guanacos. This preference for specific organs, coupled with differences in metacestode sizes between the 2 parasites, allows differential diagnosis during guanaco meat inspection. Such refined diagnostics are crucial for public health, ensuring the safety of meat products while advancing the comprehension on the role of guanacos in perpetuation of life cycle of these tapeworms. This knowledge not only could aid in the effective management of these infections but also contributes to broader epidemiological insights, facilitating improved disease control and prevention strategies in affected regions. It has been reported that the G7 genotype of *E. granulosus* appears to infect preferentially the liver (Sanchez *et al*., 2022).

The identification of *E*. *granulosus* in guanacos from both continental Patagonia and Tierra del Fuego island underscores the widespread prevalence of this zoonotic parasite in the region. The presence of *E*. *granulosus* in guanacos not only raises concerns about the potential zoonotic risk to humans and other animals but also highlights the intricate ecological interactions that facilitate the parasite's transmission across different hosts and environments.

The detection of *T. pisiformis* in a guanaco marks a rare finding that expands our understanding of the host range and ecological breadth of this parasite. While *T. pisiformis* is commonly associated with canids as definitive hosts (and rarely with felids), and lagomorphs as intermediate hosts, its presence in guanacos could indicate a broader spectrum of intermediate host species than previously recognized. Furthermore, the infection of guanaco with *T*. *pisiformis* could reflect a high level of environmental contamination with dog feces (Arona and Schiavini, 2023).

This study significantly adds further data to the understanding of *T*. *omissa*, emphasizing the importance of enhanced monitoring and precise identification of parasitic infections in wildlife, which are crucial for both veterinary public health and conservation biology. The findings highlight the potential for *T*. *omissa* to serve as an indicator of ecosystem health and predator–prey dynamics. Future research should aim to uncover the ecological factors influencing *T*. *omissa*'s distribution, delve into its genetic variability and evaluate its effects on the health and sustainability of intermediate host populations and their habitats. Such investigations will not only broaden our knowledge of *T*. *omissa*'s ecological niche but also inform conservation strategies and public health policies, ultimately contributing to the preservation of biodiversity and ecosystem resilience.

## Conclusions

Our findings reveal that *T*. *omissa* involves guanacos as intermediate hosts in Patagonia, where they represent the primary prey for pumas. The role of pumas in the life cycle of *T*. *omissa* in Magallanes remains to be clarified. We also report *T*. *pisiformis* for the first time in guanacos. Additionally, the presence of *E*. *granulosus* in guanacos accentuates the need to address the public health concerns posed by feral domestic dogs in the Patagonian regions of Chile and Argentina.

## Data Availability

Unique individual sequences of the *cox*1 gene from *T. omissa* acquired in this study were deposited in GenBank under accession numbers PP326076–PP326081.
